# Loss of Myotubularin Function Results in T-Tubule Disorganization in Zebrafish and Human Myotubular Myopathy

**DOI:** 10.1371/journal.pgen.1000372

**Published:** 2009-02-06

**Authors:** James J. Dowling, Andrew P. Vreede, Sean E. Low, Elizabeth M. Gibbs, John Y. Kuwada, Carsten G. Bonnemann, Eva L. Feldman

**Affiliations:** 1 Department of Pediatrics, University of Michigan Medical Center, Ann Arbor, Michigan, United States of America; 2 Department of Neurology, University of Michigan Medical Center, Ann Arbor, Michigan, United States of America; 3 Department of Molecular, Cellular and Developmental Biology, University of Michigan Medical Center, Ann Arbor, Michigan, United States of America; 4 Division of Pediatric Neurology, Children's Hospital of Philadelphia, Philadelphia, Pennsylvania, United States of America; The Jackson Laboratory, United States of America

## Abstract

Myotubularin is a lipid phosphatase implicated in endosomal trafficking *in vitro*, but with an unknown function *in vivo*. Mutations in myotubularin cause myotubular myopathy, a devastating congenital myopathy with unclear pathogenesis and no current therapies. Myotubular myopathy was the first described of a growing list of conditions caused by mutations in proteins implicated in membrane trafficking. To advance the understanding of myotubularin function and disease pathogenesis, we have created a zebrafish model of myotubular myopathy using morpholino antisense technology. Zebrafish with reduced levels of myotubularin have significantly impaired motor function and obvious histopathologic changes in their muscle. These changes include abnormally shaped and positioned nuclei and myofiber hypotrophy. These findings are consistent with those observed in the human disease. We demonstrate for the first time that myotubularin functions to regulate PI3P levels in a vertebrate *in vivo*, and that homologous myotubularin-related proteins can functionally compensate for the loss of myotubularin. Finally, we identify abnormalities in the tubulo-reticular network in muscle from myotubularin zebrafish morphants and correlate these changes with abnormalities in T-tubule organization in biopsies from patients with myotubular myopathy. In all, we have generated a new model of myotubular myopathy and employed this model to uncover a novel function for myotubularin and a new pathomechanism for the human disease that may explain the weakness associated with the condition (defective excitation–contraction coupling). In addition, our findings of tubuloreticular abnormalities and defective excitation-contraction coupling mechanistically link myotubular myopathy with several other inherited muscle diseases, most notably those due to ryanodine receptor mutations. Based on our findings, we speculate that congenital myopathies, usually considered entities with similar clinical features but very disparate pathomechanisms, may at their root be disorders of calcium homeostasis.

## Author Summary

Congenital myopathies are inherited muscle conditions typically presenting in early childhood. They are individually rare, but as a group are likely as common as conditions such as muscular dystrophy. The zebrafish is an emerging experimental system for the study of myopathies. We have utilized the zebrafish to develop a model of myotubular myopathy, one of the most severe childhood muscle diseases and a condition whose pathogenesis is poorly understood. We have generated fish that have the characteristic behavioral and histological features of human myotubular myopathy. Using this model, we then made novel insights into the pathogenesis of myotubular myopathy, including the identification of abnormalities in the muscle tubulo-reticular system. We subsequently identified similar changes in muscle from patients with myotubular myopathy, corroborating the importance of our zebrafish findings. Because a functional tubulo-reticular complex is required for normal muscle contraction, we speculate that the weakness observed in myotubular myopathy is caused by breakdown of this network. In all, our study is the first to identify a potential pathomechanism to explain the clinical features of myotubular myopathy. Furthermore, by revealing abnormalities in the tubulo-reticular system, we provide a novel link between myotubular myopathy and several other congenital myopathies.

## Introduction

Myotubular myopathy is a severe, X-linked congenital myopathy with onset in infancy [1]. It is characterized by profound neonatal hypotonia and skeletal muscle weakness. It is associated with substantial mortality, with approximately half of all affected boys dying in the first year of life [2]. Surviving children have significant morbidity associated with respiratory compromise and difficulties with ambulation. Currently there are no treatments or disease modifying therapies available for this condition.

The condition is defined by characteristic changes observed on muscle biopsy [1]. Biopsies show muscle fiber hypotrophy and an abundance of fibers with large, centralized nuclei of unusual appearance. These nuclei are distinct in appearance from those observed in degenerative conditions like Duchenne muscular dystrophy, and are the defining pathologic features of a group of congenital myopathies called centronuclear myopathies [3].

Myotubular myopathy is caused by mutations in the myotubularin gene [4]. Over 200 mutations have been reported in the myotubularin gene, the majority of which result in loss of functional gene expression [1]. Myotubularin is the only gene associated with myotubular myopathy. It is the canonical member of a large family of homologous proteins called the myotubularin related proteins (MTMRs) [5]. Of interest is the fact that several MTMRs are mutated in human neurologic diseases, including mutation of MTMR14 in an autosomal form of centronuclear myopathy [6].

Myotubularin was originally characterized as a protein tyrosine phosphatase, but was subsequently found instead to function primarily as a lipid phosphatase [7],[8]. It acts specifically to remove phosphates from the 3-position of phosphoinositide rings. As demonstrated in cell free biochemical assays [7],[8] and with forced exogenous expression [9],[10], myotubularin converts phosphoinositide-3-phosphate (PI3P) to phosphoinositide phosphate (PIP) and phosphoinositide-3,5-bisphosphate (PI3,5P2) to phosphoinositide-5-phosphate (PI5P). Most recently, Cao and colleagues have demonstrated using RNAi in A431 cells that knockdown of myotubularin results in a 60–120% increase in PI3P levels, thus substantiating the requirement for myotubularin in the regulation of endogenous PI3P [11]. Increased PI3P levels have also been observed in yeast lacking the myotubularin homolog ymr1 [8],[12]. As yet, however, this activity has not been directly examined in whole vertebrates or in specific organ systems, including muscle. The functional importance of myotubularin's phosphatase activity is assumed from the fact that missense mutations that alter critical amino acids in the phosphatase domain without affecting protein stability result in myotubular myopathy [1].

Phosphoinositides are implicated in myriad cellular functions, chief among them the regulation of membrane traffic and vesicle/organelle movement [13]. Because it acts to modify certain PI residues, myotubularin is assumed to function as a regulator of membrane traffic and in particular the movements of vesicles between endosomal compartments [14],[15]. Overexpression of myotubularin in cell culture delays traffic out of the endosomal compartment and causes vacuole accumulation. However, as with myotubularin phosphatase activity, a role for myotubularin in the regulation of membrane traffic *in vivo* and specifically in skeletal muscle has yet to be determined. In addition, unlike with other myopathies due to altered membrane traffic (examples include Danon Disease due to LAMP2 mutation [16]), myotubular myopathy is not characterized by the pathologic accumulation of vesicles.

Many critical questions remain unanswered concerning myotubularin function and myotubular myopathy pathogenesis. These include whether myotubularin truly functions as a lipid phosphatase and regulator of membrane traffic *in vivo*. Furthermore, the relationship between the proposed functions of myotubularin and disease pathogenesis is not clear. The same is true with the association between the unique histologic appearance of the muscle in myotubular myopathy patient biopsies and the etiology of muscle weakness and hypotonia. The lack of knowledge concerning these fundamental issues is a significant barrier in the development of therapeutic strategies for the disease.

A murine model of myotubular myopathy exists, generated by targeted mutagenesis [17]. It recapitulates the clinical and histopathologic features of the disease, thus confirming the association between myotubularin and myotubular myopathy. However, due in part to technical limitations with the murine system, it does not address many of the fundamental questions mentioned above. To begin answering these questions, and to develop a model system amenable to rapid testing of therapeutic strategies, we report here the development of a zebrafish model of myotubular myopathy. Using antisense morpholino technology, we generated zebrafish embryos with reduced myotubularin protein expression. These embryos have severely impaired motor function, muscle fiber atrophy and the presence of large, abnormally located nuclei. These findings are reminiscent of those seen in myotubular myopathy. We also demonstrate that loss of myotubularin causes increased PI3P levels in muscle, thus confirming for the first time that myotubularin functions as a lipid phosphatase in a vertebrate model system. Using RNA-mediated rescue experiments, we show that the homologous myotubularin-related genes MTMR1 and MTMR2 are able to functionally compensate for the loss of myotubularin. Lastly, and most significantly, we identify alterations in the T-tubule and sarcoplasmic reticular networks in morphant zebrafish muscle. We confirm that similar disorganization of the tubulo-reticular network is present in biopsy samples from patients with myotubular myopathy. In all, we have successfully created a zebrafish model of myotubular myopathy, and have used this model to both answer fundamental questions concerning myotubularin function and to uncover a novel mechanism to explain the pathogenesis of the disorder.

## Results

### Morpholino Knockdown of Zebrafish Myotubularin

To study the function of myotubularin (MTM1) in zebrafish, we employed antisense morpholinos to achieve functional gene knockdown. We first identified the zebrafish homolog of MTM1 using the Ensembl genome browser (ENSDARG00000037560). By bioinformatics and RT-PCR from zebrafish embryonic RNA, we found that MTM1 and 12 of 14 of the MTM1-related gene products (MTMRs) are expressed in the developing fish (Figure S1). We then designed morpholinos to the translation start site (ATG MO), to the splice donor site of exon 1 (Ex1 MO), and to the splice acceptor site of exon 3 (Ex3 MO). Both splice morphants were predicted to result in the loss of an exon and the introduction of a premature stop codon. These morpholinos were independently injected into 1–4 cell stage embryos and then embryos were phenotypically analyzed at 24, 48, and 72 hours post fertilization (hpf). A control morpholino (CTL MO) designed to a random sequence of nucleotides not found in the zebrafish genome was used to control for injection related non-specific effects [18].

The efficacy of the ATG morpholino to interfere with translation was verified by the demonstration of reduced myotubularin protein levels by immunofluorescence and western blot analysis of samples from ATG MO injected embryos (Figure S2A, B). The ability of the splice morphants to alter myotubularin RNA processing and stability was confirmed by RT PCR analysis using primers to flanking exons (Figure S2C). Of note, all 3 morpholinos yielded indistinguishable phenotypes. The ATG morpholino was used for analysis and quantitation in all subsequent experimentation, with all phenotypic observations additionally verified using the two splice morpholinos.

### Knockdown of Zebrafish Myotubularin Results in Abnormal Skeletal Muscle Function

**Figure 1:**
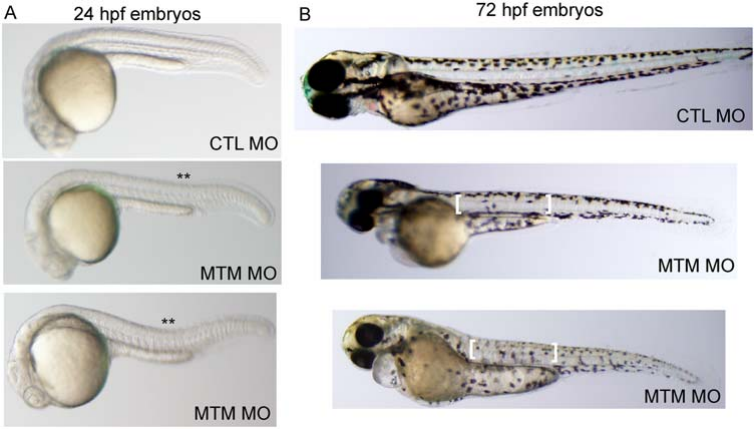
Abnormal morphology in myotubularin morphant embryos. (A) Live embryos at 24 hpf injected with either control (CTL) or myotubularin (MTM) morpholinos. MTM morphants are of equivalent size, but are bent or U-shaped in appearance. (B) Live embryos at 72 hpf injected with control (CTL MO) or myotubularin (MTM MO) morpholinos. MTM morphants are mildly dysmorphic in appearance, and display selective thinning of the muscle compartment (brackets) as well as foreshortening of their tails.

Zebrafish embryos undergo rapid skeletal muscle development, and multinucleated myotubes are present and easily recognizable by 24 hours post fertilization. We thus began our analysis at this time point. Live microscopic analysis of myotubularin morphant embryos revealed a subtle but reproducible abnormality in body shape. Specifically, knockdown embryos exhibited a dorsal curvature (**) through the back and tail instead of the normal flat or C-shaped dorsum (Figure 1A). A similar morphologic abnormality has been observed in other zebrafish models of congenital myopathies [19],[20].

Myotubularin morphant zebrafish began exhibiting more distinct morphologic abnormalities starting at 48 hpf, with the most obvious changes present in embryos at 3 days post fertilization (Figure 1B). The most consistent finding was thinning of the muscle compartment (bracket, Figure 1B). Morphant embryos also frequently had bent and/or foreshortened tails, a feature commonly associated with abnormalities in muscle structure or function (arrow, Figure 1B). Of note, the most severely affected embryos (ex: bottom embryo, Figure 1B) also exhibited changes consistent with an overall delay in embryonic development (small heads, abnormally shaped yolk balls, and reduced body extension).

**Figure 2:**
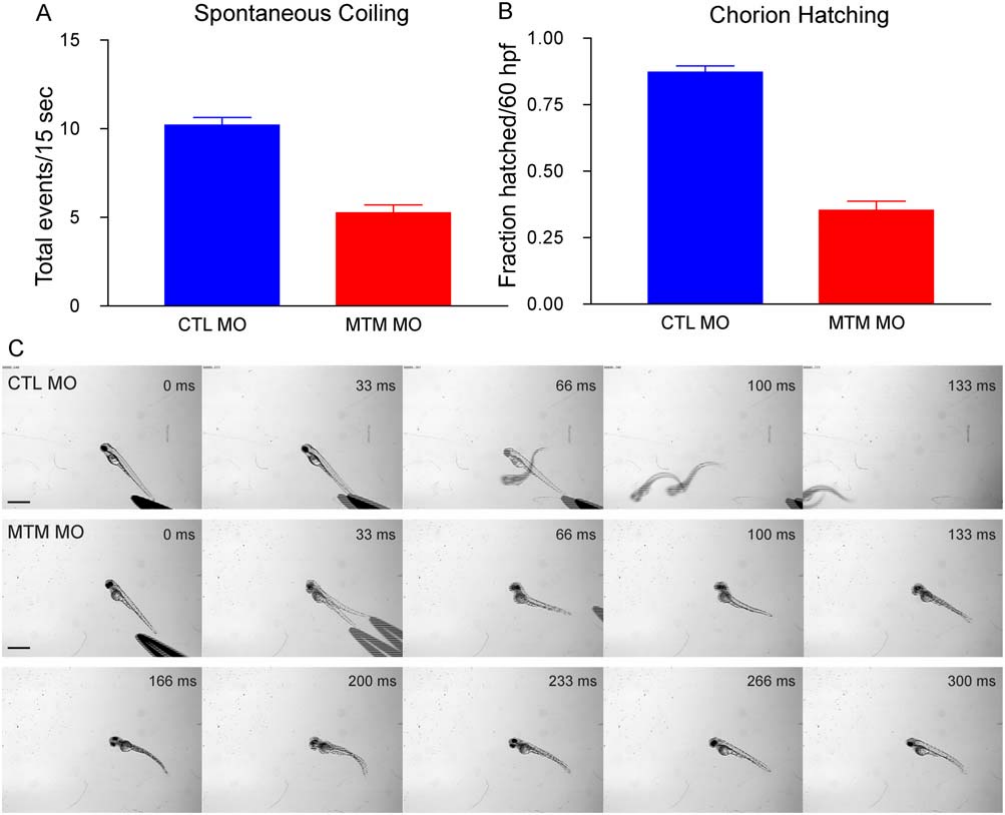
Abnormal motor function in myotubularin morphants. (A) Quantitation of spontaneous embryo coiling at 24 hpf (see also Supplemental Videos 1 and 2). On average, CTL morphants coiled 10.2 times in 15 seconds, while MTM morphants coiled only 5.2 times. (B) Quantitation of chorion hatching in 60 hpf morphants. 87.2% of CTL morphants are hatched from their protective chorions by 60 hpf, as opposed to only 35.3% of MTM morphants. (C) Touch-evoked swimming was video captured in 72 hpf morphants. As expected, CTL morphants responded to tactile stimuli with a rapid escape response contraction followed by swimming. Conversely, MTM morphants displayed a weak escape contraction followed by “twittering” movements (example at 33 ms) but never normal swimming. Scale bar = 1 mm.

In zebrafish, the first recognizable muscle dependent motor function, detected between 17 and 26 hpf, is spontaneous embryo coiling [21]. On average, control injected embryos had 10.2 (+/−0.4) spontaneous muscle contractions per 15 second period (Supplemental Video 1). Conversely, embryos injected with myotubularin morpholinos had only 5.2 (+/−0.5) contractions in the same period (Figure 2A and Supplemental Video 2). This abnormality was highly reproducible (P<0.0001), and marked the earliest observed functional abnormality in zebrafish with reduced myotubularin levels.

In addition to a decrease in spontaneous coiling frequency, myotubularin morphants also displayed defective motor behaviors later in development. Normally bouts of muscle activity contribute to the hatching of larvae from their protective outer chorion between 48 and 60 hpf. Typically approximately 90% (87.2%+/−2.3%) of control injected embryos by 60 hpf had hatched from their chorions (Figure 2B). In contrast, only 35.3% (+/−3.3%) of age-matched myotubularin morpholino-injected embryos were found to have hatched (Figure 2B), consistent with a continued decrease in muscle activity. In the most severe morphants, delayed embryonic development also likely contributed to the reduction in chorion hatching.

Once hatched, the myotubularin morphant larvae also displayed profound abnormalities in touch-evoked escape behaviors. Typically, 72 hpf larvae respond to tactile stimuli with a rapid and vigorous escape contraction, followed by swimming, which often resulted in larvae swimming out of the field of view (Figure 2C; Supplemental Video 3). In contrast, myotubularin morphants displayed weak escape contractions, followed by diminished swimming that often failed to propel the larvae out of the field of view (Figure 2C; Supplemental Video 4). The delayed chorion hatching, diminished touch-evoked escape behaviors, and morphologic changes were highly indicative of decreased muscle function.

### Myotubularin Knockdown Results in Severe Abnormalities in Muscle Structure

**Figure 3:**
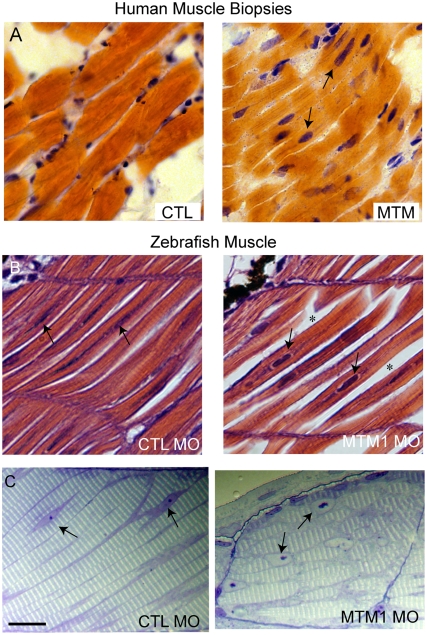
Abnormal histopathology in 72 hpf myotubularin morphants. (A) H/E stained longitudinal myofibers from myotubular myopathy (MTM) and age matched control (CTL) human muscle biopsies. Arrows point to abnormal nuclei. (B) H/E stained longitudinal myofibers from control (CTL MO) and myotubularin (MTM MO) morphant 72 hpf embryos. Myonuclei are abnormally rounded (arrows), and there is increased space between fibers (*). (C) Toluidine blue stained semi-thin sections from 72 hpf morphants. Myonuclei from myotubularin morphants are large, abnormally rounded, and contain discrete nucleoli (arrows). Sarcomeric units, however, are normal in appearance. Scale bar = 20 mm.

Severe muscle pathology, observed at both the light and electron microscopic levels, underlied the functional defects described above. We focused our analysis on muscle from 72 hpf embryos, as the muscle structure at this age is mature and greatly resembles that of human muscle. Light microscopic analysis of hematoxylin/eosin stained myotubularin morphant muscle revealed thin myofibers with abnormally located nuclei (**, Figure 3B). Analysis of semi-thin sections more dramatically illustrated these abnormal nuclei, which were mislocalized, large and filled with nucleoli of unusual appearance (Figure 3C). These findings are highly reminiscent of the nuclear abnormalities observed in human myotubular myopathy, shown in longitudinal section in Figure 3A.

**Figure 4:**
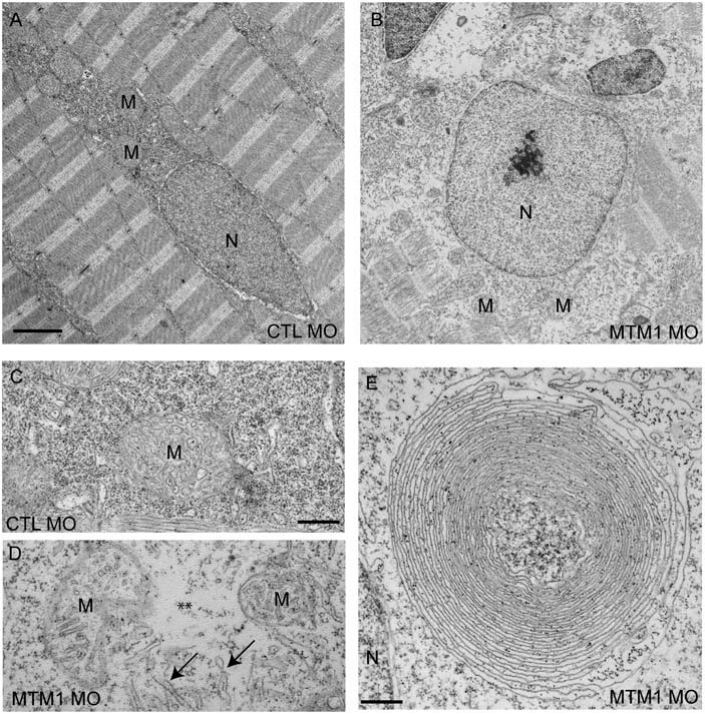
Abnormal perinuclear ultrastructure in 72 hpf myotubularin morphants. Comparison of the perinuclear area from control (CTL MO) and myotubularin (MTM MO) morphants. (A, B) Control injected embryos had thin myonuclei (N) with well organized perinuclear organelles (M = mitochondria). Myotubularin injected embryos had large, rounded nuclei (N) and disorganized perinuclear compartments. Three embryos from three independent injections were examined. Higher magnification (C, D) of the perinuclear compartment revealed abnormal mitochondria (M), areas nearly devoid of organelles (**), and several tubule-like structures (arrows). (E) Example of an unusual membranous perinuclear structure. Such structures were observed in multiple myofibers. Scale bars: A, B (2 mm), C–E (500 nm).

We further characterized the perinuclear compartment using electron microscopy (Figure 4). Nuclei from myotubularin morphants were again found to be unusual in appearance (Figure 4B). The nuclei were surrounded by enlarged areas of disorganized cytoplasm which had a relatively paucity of normally appearing organelles. Higher magnification of the perinuclear compartment underscored the perinuclear changes, revealing abnormal mitochondria, areas lacking any organellar structure, and disorganized tubule-like structures (Figure 4C) In addition, some fibers contained large, bizarre, membranous structures of unclear origin (Figure 4D). This perinuclear disorganization is commonly observed in human myotubular myopathy muscle biopsies, and similar membranous structures have also been reported [22]. Of note is that we did not observe obvious vacuoles in the perinuclear area of any myofibers examined, which is contrary to what might be expected for a defect in endosomal trafficking.

### Myotubularin Morphants Exhibit Myofiber Hypotrophy

**Figure 5:**
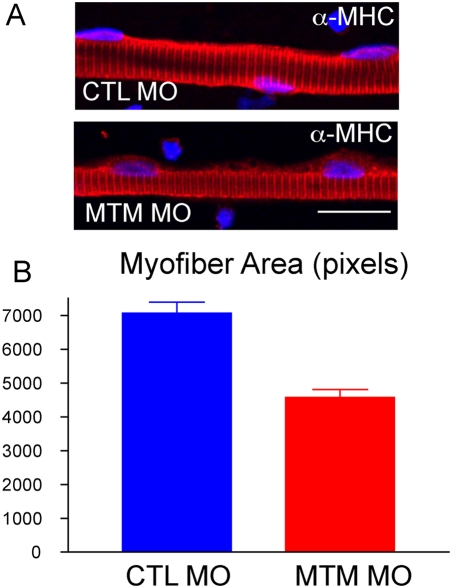
Myofiber hypotrophy in myotubularin morphants. (A) Representative myofibers from control (CTL) and myotubularin (MTM) morphant embryos at 72 hpf. Fibers were immunostained with an antibody to myosin heavy chain (a-MHC). MTM fibers have normal MHC staining, but appear thinner. Scale bar = 20 mm. (B) Quantitation of myofiber size. Control myofibers averaged 7000 pixels, while myotubularin morphant fibers were only 4000 pixels.

The fact that myotubularin morphants had thin appearing muscle compartments by live image analysis (Figure 2) suggested that the muscle fibers may be hypotrophic as compared to controls. To examine this, we isolated myofibers from 72 hpf control and myotubularin morpholino injected embryos. Myofiber size was determined by calculating the area of myofibers stained by immunofluorescence with a myosin heavy chain (MHC) antibody. Myofibers from myotubularin morphants were significantly smaller than those from controls, measuring only 50% of control area (Figure 5A, B). The reduced size was not due to loss of myofiber structural integrity, as evidenced by the normal appearance of sarcomeric structures with MHC antibody labeling. Myofiber hypotrophy is an abnormality that is commonly observed in the muscle from myotubular myopathy patients [2].

### Myotubularin Regulates PI3P Levels in Skeletal Muscle *In Vivo*

One of the central questions related to myotubularin function is whether it has lipid phosphatase activity *in vivo*. To address this, we measured levels of PI3P, the primary lipid upon which myotubularin acts *in vitro*, in morpholino-injected embryos. For whole embryo measurements, we extracted total lipids and then used a lipid-protein-antibody overlay technique. When normalized to PI4P levels, the amount of PI3P detected in lipid preps from myotubularin morphants was not significantly different from the level in controls (Figure S3). The fact that overall PI3P levels were not changed was unsurprising considering that 7 other MTMRs with PI3P phosphatase activity are present in the fish embryo (see Figure S1).

**Figure 6:**
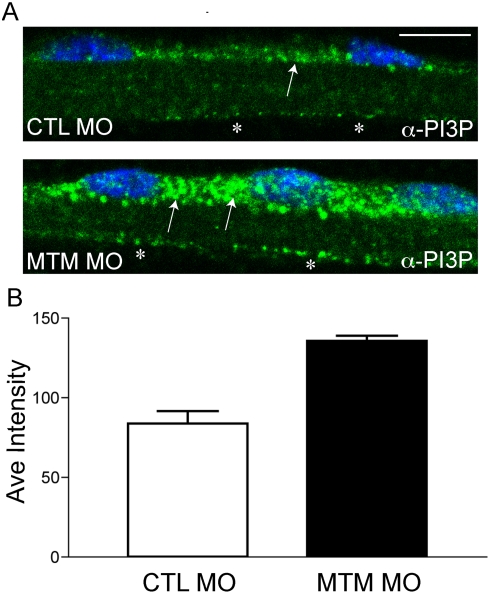
Increased PI3P levels in myotubularin morphant myofibers. (A) Representative myofibers immunostained with anti-PI3P. Perinuclear staining of PI3P in myotubularin morphant myofibers is much more abundant than in control myofibers (arrows). There is also a modest increase in membrane localized PI3P (*). Scale bar = 10 mm. (B) Quantitation of PI3P immunofluorescence. PI3P intensity measured over a uniform perinuclear area (see methods for details) and was 83.7+/−7.8 pixels for control morphants and 135.5+/−3.3 for myotubularin morphants (3 trials; p = 0.0027). This represented a 1.6× increase in PI3P staining intensity.

Given that myotubularin is specifically required for muscle function, we next wanted to measure PI3P levels in muscle only. To accomplish this, we performed quantitative immunofluorescence on isolated myofibers using a PI3P antibody. Myotubularin morphant myofibers had readily observable increases in PI3P antibody staining, in particular in the perinuclear area (Figure 6A). We quantitated the pixel intensity of the perinuclear PI3P staining, and found that myotubularin morphants had levels 1.6 times higher than observed in controls (Figure 6B). This was consistent with a loss of myotubularin's phosphatase activity in the muscle, and provided evidence that myotubularin functions to regulate PI3P levels in muscle *in vivo*.

### MTMRs 1 and 2 Compensate for the Loss of Myotubularin

**Figure 7:**
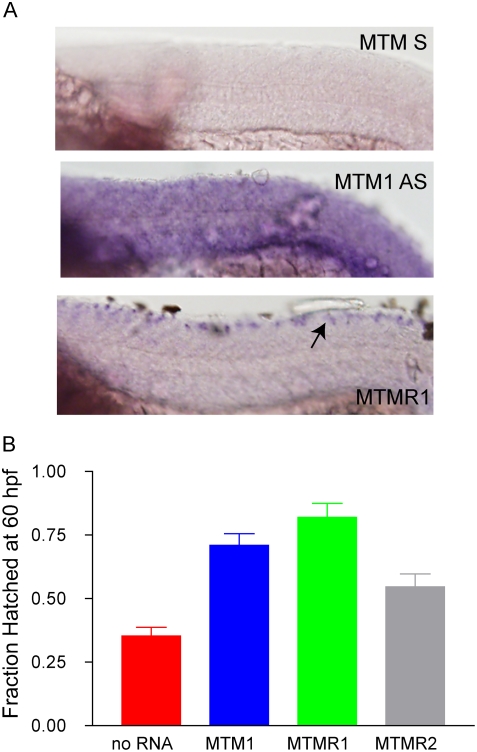
MTMR rescue of the myotubularin morphant phenotype. (A) Whole mount in situ hybridization of 24 hpf embryos reveals muscle staining for myotubularin (MTM1 AS) and not for MTMR1 or MTMR2 (data not shown). A sense probe to MTM1 (MTM1 S) was used as a background control. (B) RNA rescue experiment. RNA to MTM1, MTMR1 or MTMR2 was co-injected with myotubularin morpholino. Rescue was determined by the % of hatched embryos at 60 hpf. Values were: No RNA (35.5%+/−3.3%, N = 201), MTM1 RNA (71%+/−4.5, N = 100, p (when compared to No RNA)<0.0001), MTMR1 RNA (82%+/−5.5%, N = 50, p<0.001), MTMR2 RNA (54.6%+/−5.1% N = 97, p = 00015).

A potential explanation for the fact that PI3P levels are normal in the whole embryo but increased in muscle is that myotubularin is the sole or primary PI3P phosphatase in muscle while other MTMRs are expressed in other tissues. This question has been examined in murine myocytes by RT-PCR, and myotubularin was found to be the predominant phosphatase expressed in differentiated fibers [23]. We examined this question in the developing zebrafish using whole mount RNA *in situ* hybridization. We focused on the expression of myotubularin and its two most closely related homologs, MTMR1 and MTMR2. We found that between 24 hpf and 72 hpf, only myotubularin was expressed in muscle (Figure 7A and data not shown), supporting the idea that it is the primary PI3P phosphatase in that tissue.

We thus hypothesized that myotubularin knockdown results specifically in abnormalities in muscle because other functionally similar MTMRs are not expressed in muscle. To test this, we performed a series of gene rescue experiments (Figure 7B). We injected embryos with myotubularin morpholino and RNA from either myotubularin, MTMR1, or MTMR2 and measured the ability of embryos to hatch from their chorions by 60 hpf. In these experiments, the morpholino and the RNA are expressed ubiquitously. As expected, injection of morpholino alone caused a significant reduction in hatching (35% hatched; see also Figure 2B), while co-injection with full-length zebrafish myotubularin RNA resulted in significant amelioration of this hatching defect (71% hatched). Interestingly, co-injection of morpholino with either MTMR1 or MTMR2 RNA also restored the ability to hatch from the chorion. MTMR1 rescued hatching nearly to control levels (82%hatched; Figure 7B), while MTMR2 resulted in more modest improvement (55% hatched; Figure 7B). Therefore, these functionally similar MTMRs can compensate for the lack of myotubularin function in skeletal muscle.

### Myotubularin Knockdown Results in T-Tubule Abnormalities

**Figure 8:**
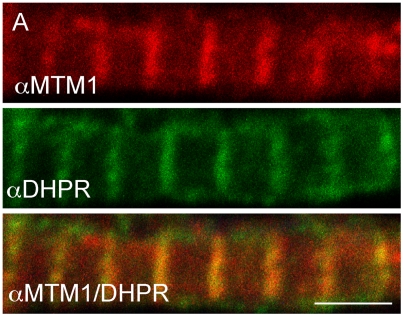
Myotubularin localizes to T-tubules. Myotubularin and DHPRa1 co-localize. Double label immunofluorescence was performed on isolated myofibers. As demonstrated using confocal microscopy, myotubularin (red) and DHPRa1 (green) signal significantly overlap (orange, panel 3). Scale bar = 10 mm.

A recent study on mouse myotubularin by Buj-Bello and colleagues reported localization of the protein to the T-tubule/sarcoplasmic reticulum junction [24]. We examined myotubularin subcellular localization in zebrafish myofibers, and determined by immunofluorescent analysis that the protein was expressed in a distinctive linear pattern that overlaps with that of the dihydropyridine receptor (DHPR), a marker for T-tubules (Figure 8). This pattern is thus consistent with localization to T-tubules. As expected, this staining was essentially undetectable in myofibers derived from myotubularin morphants (Figure S2).

**Figure 9:**
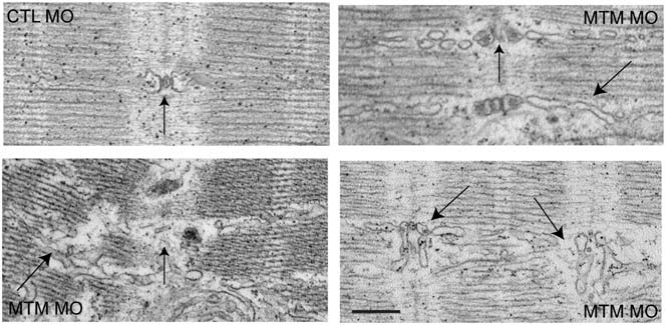
T-tubule structural abnormalities in myotubularin morphant muscle. T-tubule (vertical arrows) and sarcoplasmic reticulum (angled arrows) abnormalities as demonstrated by electron microscopy. Control morphant (panel 1; CTL): normal T-tubule triad with accompanying thin, well-organized SR network (arrow). Myotubularin morphants (panels 2–4): Panel 2 shows mildly dilated triads and SR networks. Panel 3 shows severely dilated and dysmorphic triads and widely looped SR. Panel 4 illustrates severe disorganization, with unrecognizable T-tubule triads and aberrant adjacent SR networks. Scale bar = 500 nm.

Based on this localization, we were interested in the effect of myotubularin knockdown on T-tubule organization. We performed ultrastructural analysis using electron microscopy (Figure 9). Muscle from control morpholino injected embryos exhibited the normal pattern of T-tubules and sarcoplasmic reticulum (SR), with the SR coursing tightly through the sarcomeres and the T-tubules forming triads at regular periods. Conversely, muscle from myotubularin morpholino injected embryos had grossly aberrant SR and T-tubule networks (Figure 9). The SR networks were irregular, disorganized, and often randomly interspersed throughout the sarcomere. The T-tubule triads showed a range of abnormalities, from mild changes in electron density of the triad (arrow, upper right panel), to severe dilation of the triad structure (arrows, lower right panel), to fibers with essentially unrecognizable SR/triad areas (arrow, lower left panel).

We next determined if these ultrastructural changes corresponded to alterations in T-tubule function. We focused on excitation-contraction coupling, a process that requires intact T-tubules. We first verified that nervous system output to muscle was normal by assaying touch-evoked fictive swimming. To examine this, whole-cell voltage recordings were made from myofibers *in vivo*. In both control and myotubularin morphants, tactile stimulus resulted in rhythmic membrane depolarization in skeletal muscle (Figure S4). These data are consistent with intact output from the nervous system and through the neuromuscular junction [25],[26].

**Figure 10:**
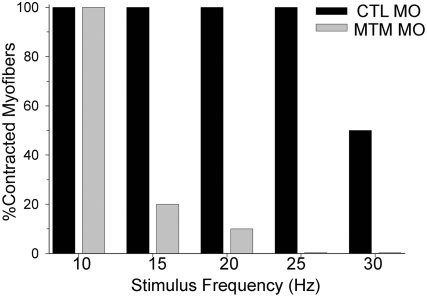
Excitation-Contraction Coupling abnormalities in myotubularin morphants. Excitation-contraction (E–C) coupling. Control morphant myofibers could respond to 15 ms depolarizing current injections to 0 mV from 0 to 30 Hz (average max frequency = 27.0+/−0.9 Hz, N = 5). In contrast, myotubularin morphant muscle progressively failed to contract to stimuli above 10 Hz (average maximum frequency = 11.5+/−1.5 Hz, N = 5, p<0.0001).

We then proceeded to study excitation-contraction coupling (Figure 10). This was accomplished by measuring the ability of myofibers to contract when exposed to depolarizing stimuli of progressively higher frequencies [27]. Employing this technique, we found that control myofibers consistently contracted at all stimuli up to 30 Hz, with the average maximal frequency equaling 27.0 Hz. Conversely, myofibers from myotubularin morphants exhibited increasing abnormalities above 10 Hz, with no myofibers able to contract to stimuli at 25 Hz and the average maximal frequency equaling only 11.5 Hz. This result is consistent with a defect in excitation-contraction coupling, and provides functional evidence to support the morphologic abnormalities observed in the T-tubules.

### Myotubular Myopathy Muscle Biopsies Exhibit T-Tubule Disorganization

**Figure 11:**
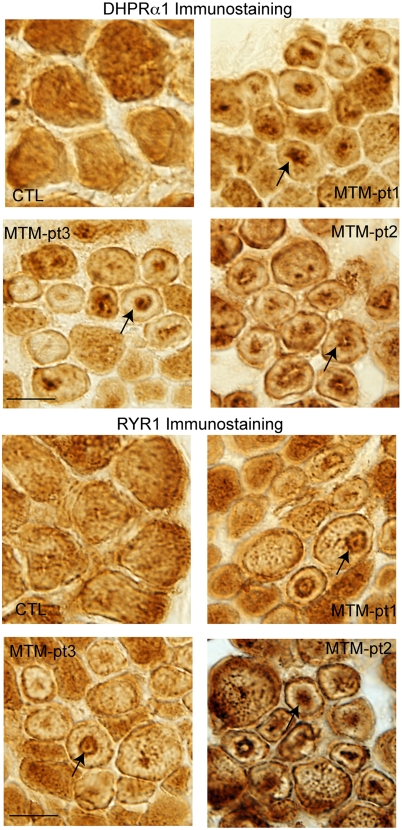
Alteration of T-tubule/SR component localization in myotubular myopathy. Muscle from 3 myotubular myopathy patients and an age matched control were immunostained with DHPRa1 to mark T-tubules (A) and RYR1 to mark the adjacent sarcoplasmic reticulum (B). Abnormal distribution of DHPRa1 and RYR1 was observed in numerous fibers in all 3 myotubularin patients but in none of the control fibers (arrows). Scale bar = 20 mm.

We were interested to correlate our findings with muscle from myotubular myopathy patient biopsies. T-tubule abnormalities have not been specifically mentioned in previous pathologic analyses from myotubular myopathy patients. We examined T-tubule organization in human biopsy samples using immunohistochemistry and antibodies to DHPRa1, a T-tubule marker, and to RYR1, a marker of the adjacent sarcoplasmic reticulum. A similar technical approach was successfully utilized by Laporte and colleagues to examine T-tubule organization in centronuclear myopathy patients with BIN1 mutations [28]. As a staining control, we used muscle from an unaffected, age matched control sample. DHPRa1 and RYR1 staining in the control muscle were found in the expected pattern along the membrane and throughout the cytoplasm (Figure 11A and Figure 11B, respectively). Conversely, samples from three patients revealed clear abnormalities in both DHPR and RYR1 staining patterns. T-tubules were found concentrated around the abnormally located central nuclei, or in irregular densities in the centers of several fibers. Importantly, other plasma membrane components were not found in this distribution (Figure S5), indicating that this disorganization is relatively specific for T-tubules.

**Figure 12:**
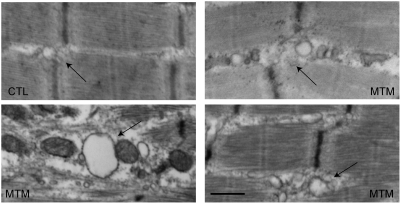
Ultrastructural changes in T-tubules in myotubular myopathy. Electron microscopic analysis of muscle from 3 myotubular myopathy patients (MTM) and one age-matched control (CTL). Control T-tubule triads are discretely formed (arrow), and the adjacent SR network is thin and well organized. Triads and adjacent SR from patient biopsies are dilated and disorganized. Scale bar = 500 nm.

We lastly examined electron micrographs obtained from patient muscle biopsies (Figure 12). Age matched control muscle showed the typical tight triad structure with well-organized adjacent sarcoplasmic reticulum. In contrast, micrographs from 3 myotubular myopathy patients showed various degrees of dilatation and disorganization of the T-tubules and adjacent sarcoplasmic reticulum. In conjunction with the immunostaining, these data confirm that T-tubule abnormalities are present in both our zebrafish model and in patients with myotubular myopathy.

## Discussion

We used antisense morpholinos to investigate the effect of myotubularin knockdown on zebrafish development. Our data from these studies illuminate several novel insights into myotubularin function/dysfunction. The first is that knockdown of zebrafish myotubularin recapitulates the features of myotubular myopathy, and thus demonstrates that zebrafish are an excellent model for studying the disease. The second is that closely related MTMRs can functionally compensate for the loss of myotubularin, suggesting that homolog upregulation is a viable therapeutic strategy in myotubular myopathy. The third is that T-tubule abnormalities are present in both zebrafish and human patients lacking myotubularin. As discussed below, T-tubule abnormalities are a unifying pathologic feature shared now by several congenital myopathies.

### Zebrafish as a Disease Model of Myotubular Myopathy

Myotubular myopathy is characterized clinically by the early onset of weakness and hypotonia, and pathologically by Type I fiber hypotrophy and the presence of centralized nuclei with abnormal appearance surrounded by areas of sarcoplasmic disorganization [1]. Zebrafish with reduced levels of myotubularin share all of these essential disease features. Embryos have defects in the earliest muscle dependent functional processes, including diminished spontaneous contractions and an inability to hatch from their chorions. The histopathology of myotubularin morphant fish closely mirrors the appearance of human myotubular myopathy muscle. Fibers are small (50% of control size) and have large, unusual and mislocalized nuclei surrounded by areas of sarcoplasmic disorganization. The perinuclear area also often contains accumulation of abnormal membranous structures; such structures have been reported in human ultrastructural analyses [22].

The myotubularin morphant zebrafish described here are now the second model system that recapitulates the “clinical” and pathologic features of myotubular myopathy by knocking down myotubularin levels during development. The other model is a mouse gene knockout generated by Buj-Bello, Laporte, Mandel and colleagues [17]. One interesting difference between our model and the knockout mice is the timing of the muscle phenotype. Our phenotype is present at a very early time point (essentially when primary myogenesis is completed), whereas the knockout mice have a period of normal development followed by precipitous degeneration. It is not clear which more accurately reflects the human disease, for while patients often have symptoms at birth, the ability to measure/detect *in utero* abnormalities in muscle function is difficult [1]. The difference between the two models may be reflective of the rapid and compacted development of the zebrafish. Conversely, it may be due to the fact that muscle maturation in the mouse continues for the first several postnatal weeks. Thus the difference may reflect the specifics of muscle development in the two organisms instead of intrinsic differences in myotubularin function in the species.

Our zebrafish model joins a growing list of myopathies and dystrophies that are successfully modeled in zebrafish [19], [20], [25], [29]–[32]. Given the large number of offspring that can be studied and the highly reproducible nature of our morphant phenotype, the myotubular myopathy zebrafish should provide an excellent springboard for high throughput testing of small molecule therapeutics.

### Myotubularin Regulates PI3P Levels *In Vivo*

One of the fundamental questions regarding myotubularin function was whether it behaved as a lipid phosphatase *in vivo*. We were able to address this question using our zebrafish model. Using quantitative immunohistochemistry, we demonstrate that PI3P, the principal substrate for myotubularin phosphatase activity, accumulates in myofibers from myotubularin morphant embryos. This is the predicted result from loss of myotubularin expression if it acts as a 3-position phosphatase. Significantly, these data are very consistent with the previously reported changes in PI3P levels found when myotubularin protein levels are reduced in cell culture or in yeast. We observed a 1.6 fold increase in PI3P in skeletal muscle, while Cao et al detected a 1.6 to 2 fold increase using RNAi in A431 cells and Dixon and colleagues found a 2 fold increase in ymr1 null yeast. Of note, our results represent one of the first assessments of potential phosphatase activity for any myotubularin family member *in vivo* and the first specifically in muscle.

### Functional Compensation by Homologous MTMRs

Including myotubularin, 15 MTMRs are present in the vertebrate genome. All are expressed in zebrafish, mouse and man. Eight of the 15 have apparently identical phosphatase activity, with the remaining 7 are considered “phosphatase-dead” MTMRs [33]. Because myotubularin mutations result in severe muscle disease, it seems clear that none of the phosphatase active MTMRs compensate in myotubular myopathy patients [14]. It was not known whether this is due to unique non-phosphatase properties of myotubularin, or rather due to expression differences between MTMRs. Our data convincingly support the later conclusion. We show that MTMR1 and MTMR2, the MTMRs with the highest homology to myotubularin, are not expressed in zebrafish muscle. Furthermore, exogenous ubiquitous expression of either gene rescued the myotubularin morpholino phenotype. Importantly, expression of these MTMRs in control fish did not result in obvious phenotypic abnormalities. This implies that increasing the expression of either MTMR1 or MTMR2 in patient muscle is a viable potential treatment strategy for myotubular myopathy.

### T-Tubule Abnormalities Due to Loss of Myotubularin in Zebrafish and Man

Perhaps the most significant finding from our study is that decreasing myotubularin expression or function results in both structural and functional abnormalities in the T-tubule network. This finding is significant for several reasons. The first is that it provides the first viable explanation for why patients (and mice and zebrafish) have significant weakness. T-tubules are critical for several aspects of muscle contractions and force generation, in particular for excitation-contraction coupling [34]. Impairment of this network should clearly lead to diminished force production and muscle weakness. We demonstrate this functionally in the zebrafish, as embryos with decreased myotubularin have excitation-contraction coupling abnormalities.

A second significance to these data is that they provide a plausible hypothesis for myotubularin function in myofibers. T-tubules biogenesis and maintenance is dependent on the continuous recycling of its membranous contents [15]. Membrane recycling is in turn dependent on tight regulation of phosphoinositides. Therefore, one possible explanation for our results is that myotubularin functions to regulate the recycling of T-tubule membrane components via its ability to participate in the regulation of phosphoinositide levels.

An association between T-tubule homeostasis and myotubularin is especially attractive given the potential functional similarities between T-tubule recycling and endosomal dynamics. Previous studies have shown that both endosomes and T-tubules share similar structural and regulatory components. Most notably, BIN1/amphiphysin2 and dynamin-2 are critical regulators of membrane trafficking at the endosome [35],[36], and both are expressed at the T-tubule [28]. In addition, BIN1 is required for T-tubule biogenesis in cultured myocytes and for T-tubule organization in Drosophila [37],[38]. As discussed below, mutations in both BIN1 and dynamin-2 result in centronuclear myopathy [1], a myopathy with similar pathologic features to myotubular myopathy. Such overlapping roles are also seen with caveoli, which are critical for both T-tubule formation/maintenance and for endocytosis [39],[40]. Thus, given the many observations functionally linking T-tubule dynamics and the regulation of endosomes, it seems very likely that myotubularin's primary function in muscle is controlling T-tubule dynamics in a fashion analogous to that described for its regulation of endosomal trafficking *in vitro* [10],[11].

A final importance relates to other congenital myopathies. Traditionally, congenital myopathies are considered a group of independent conditions, distinguished by their histopathologic features on muscle biopsy. However, they are in general similar in clinical presentation, characterized by neonatal hypotonia and non-progressive weakness [41]. The discovery of T-tubule abnormalities in myotubular myopathy now pathogenically links the three most prevalent groups of congenital myopathies. Core myopathies are caused by mutations in the ryanodine receptor (RYR1) [42], the calcium channel located at the T-tubule/sarcoplasmic reticulum border that is required for excitation-contraction coupling [25], and by mutations in Selenoprotein-N [43], a modifier of RYR1 [20]. Most nemaline myopathies are caused by mutations in the components of the thin filaments, proteins which function downstream of RYR1 and calcium release to initiate contraction [44]. Along with the centronuclear myopathies due to BIN1 (where T-tubule abnormalities have already been documented) and dynamin-2 mutation [1], myotubular myopathy likely is an “upstream” defect, resulting in abnormalities in the underlying T-tubule and sarcoplasmic reticular structure upon which RYR1 function is dependent.

In light of this commonality between congenital myopathies, the next important step is to see if modifiers of excitation-contraction coupling and T-tubule function can ameliorate the muscle weakness in the relevant disease models. We are currently at work developing and testing such agents in our zebrafish model of myotubular myopathy.

### Summary

We have developed a new vertebrate model of myotubular myopathy, which has allowed us to answer fundamental questions regarding myotubularin function, and to make novel insights into the pathogenesis of the human disease. In the future, this model may provide a valuable platform for developing and testing novel therapeutics based on our new insights.

## Materials and Methods

### Morpholinos

Morpholinos were designed to the putative ATG, to the exon 1 splice donor site, and to the exon 3 splice acceptor site of the zebrafish myotubularin gene (GeneTools, LTC). The control morpholino (GeneTools) was designed to random sequence with no homology by BLAST analysis in the zebrafish genome. The morpholino sequences are as follows:

Control morpholino (CTL MO): CCT CTT ACC TCA GTT ACA ATT TAT A
ATG morpholino (ATG MO): AGACCCTCGTCGAAAAGTCATAACG
Exon 1 morpholino (Ex1 MO): GGAAATGCTCGGGCCTACCTTTACG
Exon 3 morpholino (Ex3 MO): CCTGTCAACACACGCAGGAACATTG

### Injections

1.5 nL of morpholino (0.08 mM) was injected into the yolk of 1–4 cell stage zebrafish embryos as described previously [18]. Embryos were subsequently grown in egg water and then analyzed at various time points. Western blot and RT-PCR analysis for determining morpholino efficacy were described previously [18].

### Live Imaging

Embryos were examined by live image analysis using a Leica stereomicroscope and camera. Both photomicrographs and videos were obtained using this system.

### Spontaneous Embryo Coiling

To measure spontaneous coiling, embryos were manually dechorionated at 24 hpf and recorded for 15 seconds. Records were obtained approximately 5 minutes after dechorionation.

### Touch Evoked Swimming

Touch-evoked motor behaviors were elicited by touching the head, yolk sac or tail with a pair of No. 5 forceps. Motor behaviors were recorded by video microscopy (∼20×) using a Panasonic CCD camera (wv-BP330) mounted on a Leica dissection microscope. Videos captured (30 Hz) on a Macintosh G4 computer with a Scion LG-3 video card using Scion Image software (www.scioncorp.com) were processed with ImageJ.

### Histopathology

For hematoxylin/eosin sections, 72 hpf embryos were fixed overnight at 4°C in 4% paraformaldehyde, washed in PBS, dehydrated in alcohols and xylenes, and embedded in paraffin. Microtome sections were cut at 2 mm. H/E was done per standard protocol. For semi thin sections and electron microscopic analysis, 72 hpf embryos were fixed overnight at 4°C in Karnovsky's fixative and then processed for sectioning by the Microscopy and Imaging Laboratory (MIL) core facility. Semi-thin sections were stained with Toludine blue. Light microscopy was performed using an Olympus BX-51 microscope and images captured with an Olympus DP-70 digital camera. Electron microscopy was performed using a Phillips CM-100 transmission electron microscope.

### Myofiber Cultures

Mixed cell cultures from 72 hpf embryos were obtained as follows. Embryos were euthanised with tricaine and the dissociated in 10 mM collagenase type I (Sigma) for 60–90 min at room temperature. Embryos were triturated approximately every 30 min. Dissociated preps were pelleted by centrifugation (5 min at 3000 rpm), resuspended in CO_2_ independent media (Invitrogen), passed through a 70 mm filter (Falcon), and plated onto chamber slides (Falcon) precoated with poly-L-Lysine (Sigma). Culture media was changed after one hour, after which cells were fixed for 15 min in 4% paraformaldehyde.

### Myofiber Immunofluorescence

Fixed cells were blocked in 10%NGS/0.3% Triton, incubated in primary antibody overnight at 4°C, washed in PBS, incubated in secondary antibody, washed in PBS, then mounted with ProLong Gold plus DAPI (Invitrogen). For PI3P antibody staining, cells were processed according to manufacturers protocol (Echelon Biosciences). The following primary antibodies and dilutions were used: mouse anti-myosin heavy chain (MF20; 1∶20; Developmental Hybridoma Bank); mouse anti-PI3P (1∶100; Echelon); rabbit anti-myotubularin (1∶50; Stratagene); and mouse anti-DHPR1a (1∶200; Abcam). Alexafluor conjugated secondary antibodies were used at 1∶250 (Invitrogen). Images were obtained by confocal microscopy as described previously [18].

### Myofiber Area

Myofiber area was measured from photomicrographs using Metamorph software. Myofibers were outlined using the freehand tool and analyzed for total two-dimensional area.

### Quantitative Immunofluorescence

PI3P antibody staining was performed as described above. Samples were analyzed on an Olympus IX-71 inverted confocal microscope and images captured using the FluoView v4.3 software. Fluorescent images were processed for quantitation using Metamorph (Sunnyvale, CA). Identical regions (immediate perinuclear area) were selected from each fiber using the rectangle tool set to a constant area. Boxed areas were then analyzed for pixel intensity. 15 myofibers from control and myotubularin morphant myofibers were compared for each region (5 per single myofiber prep×3 independent preps). Statistical significance was determined using a Students one-tailed t-test (Prism software) [45].

### Lipid Overlay Assay

The lipid overlay assay was performed per manufacturer protocol for PI3P and PI4P on lipids extracted from 72 hpf embryos (Echelon Biosciences) [11].

### Whole-Mount *In Situ* Hybridization

*In situ* hybridization was performed as described previously [18]. Probes were made by *in vitro* transcription from zebrafish cDNA plasmids (all clones obtained from Open Biosystems).

### RNA Rescue

RNA for morpholino rescue was prepared by *in vitro* transcription using the mMessage mMachine kit (Ambion). RNA was co-injected with morpholino at a concentration of 100 ng/ml. Rescue was determined by measuring the percentage of embryos hatched from their chorions at 60 hpf.

### *In Vivo* Electrophysiology

For *in vivo* electrophysiological measurements [46], larvae (72–80 hpf) were pinned in a Sylgard-coated petri dish (Dow Corning, Midland, MI) containing extracellular recording solution with curare [in mM:134 NaCl, 2.9 KCl, 2.1 CaCl2, 1.2 MgCl2, 10 glucose, 10 HEPES, pH 7.8 and 3 µM d-tubocurarine]. Larvae were manually skinned on one side, exposing muscle tissue. Electrodes were pulled from borosilicate glass and filled with internal recording solution [in mM: 116 K-gluconate, 16 KCl, 2 MgCl2, 10 HEPES, 10 EGTA, at pH 7.2 with 0.1% Sulforhodamine B for muscle cell type identification]. Whole-cell recordings were performed on individual adaxial myocytes using an Axopatch 200B amplifier (Axon Instruments, Union City, CA), low pass filtered at 1 kHz and sampled at 2–10 kHz. For each patch-clamped myocyte, steps of depolarizing current (3–6 nA) were injected to induce contraction. Current pulses were first delivered at a frequency of 1 Hz for 10 s. Frequency was increased by 5 Hz intervals until the myocyte reached tetanus. Contractions were recorded by video imaging and data acquired using a Digidata 1322A interface controlled by pClamp 8 software (Axon Instruments). Data analysis was performed using Clampfit 10.

### Touch-Evoked Fictive Swimming

Touch-evoked fictive swimming was elicited with a fire-polished recording electrode (∼50 µm) controlled by a Burleigh PCS-1000 piezoelectric manipulator and PCS-250 patch clamp driver (EXFO Life Sciences) as described previously [47] until fictive swimming was evoked.

### Section Immunohistochemistry

Cryosections from human muscle biopsies were incubated overnight at 4°C in primary antibody (DHPRa1, 1∶200; RYR1, 1∶100; α-dystroglycan, 1∶50), washed in TBS, and then processed using the kit (Novacastra). Photomicrographs were obtained on an Olympus XL.

### Western Blot Analysis

Western blot analysis was performed as previously described [48]. Rabbit anti-myotubularin (Stratagene) was used at 1∶1000 and anti-rabbit secondary (Santa Cruz Biotech) at 1∶2000. Goat anti-actin (Santa Cruz Biotech) was used at 1∶1000 and anti-goat secondary (Santa Cruz Biotech) at 1∶200. Luminenscent detection was performed using the Lumiglo reagent (Cell Signalling).

### Ethics Statement

All animals were handled in strict accordance with good animal practice as defined by national and local animal welfare bodies, and all animal work was approved by the appropriate committee (UCUCA #09835).

## Supporting Information

Figure S1 RNA expression of zebrafish MTMRs. RNA was isolated from 24 hpf zebrafish embryos and processed for RT-PCR. PCR was performed with primers specific for MTM1 and MTMRs 1–15 (excluding MTMRs 4 and 11). Bands were detected for all MTMRs tested.
(0.80 MB TIF)

Figure S2 Morpholino knockdown of zebrafish myotubularin. (A) Immunohistochemistry using a myotubularin antibody on isolated myofibers from embryos injected with control (CTL MO) or myotubularin ATG (ATG MO) morpholinos. The linear staining pattern observed in control myofibers (see Figure 8) was barely detectable in MTM1 morphant fibers. (B) Western blot analysis from protein isolated from 72 hpf embryos injected with control (CTL MO) or myotubularin ATG (ATG MO) morpholinos. A band corresponding to myotubularin was detected in CTLs but not in MTM morphants. Blots were re-probed with actin to assure equal loading. (C) RT-PCR analysis from RNA extracted from 48 hpf control (CTL), exon 3 specific (Ex3), and exon 1 specific (Ex1) myotubularin morphants. Top panel: dynamin-2 (DNM) specific primers reveal intact RNA and equal starting cDNA from all samples. Middle panel: primers spanning exons 1–3 reveal reduced products for both the exon 1 and exon 3 splice blocking morphants. Bottom panel: primers spanning exons 2–4 reveal substantially reduced product in the exon 3 splice blocking morphants but a normal product in the exon 1 morphants. Taken together, the RT-PCR data reveals that the Ex1 morpholino successfully results in exclusion of exon 1 from the final RNA. The Ex3 morpholino causes exclusion of exon 3 as well as overall reduction in MTM1 RNA, likely due to nonsense mediated decay mechanisms.
(5.31 MB TIF)

Figure S3 Total PI3P levels are unaffected in myotubularin morphants. PI3P mass strip assay performed on lipids isolated from control (CTL MO) or myotubularin (MTM MO) 72 hpf morphant embryos. No difference in PI3P levels was observed in MTM1 morphants (CTL 0.87 Pmol+/−0.10; MTM 0.83 Pmol+/−0.07; n = 3). Equal spotting of lipids was determined by measuring PI4P levels on duplicate samples (data not shown).
(1.22 MB TIF)

Figure S4 Touch-evoked fictive swimming. Touch-evoked fictive swimming in control morphants (left) and myotubularin morphants (right) in response to a 50 ms tactile stimulus. Normal responses were detected in the myotubularin morphants, indicating that neuronal input to skeletal myofibers is intact.
(0.15 MB TIF)

Figure S5 α dystroglycan staining on human biopsy samples. Immunohistochemistry with an α dystroglycan antibody. Note the normal staining around the plasma membrane and the lack of internal staining. * indicate examples of fibers with central nuclei. Scale bar = 20 mm.
(9.71 MB TIF)

Video S1 Spontaneous coiling in a 24 hpf control morphant embryo.
(4.98 MB AVI)

Video S2 Spontaneous coiling in a 24 hpf myotubularin morphant embryo.
(5.01 MB AVI)

Video S3 Touch evoke escape response in a 72 hpf control morphant embryo.
(0.93 MB AVI)

Video S4 Touch evoke escape response in a 72 hpf myotubularin morphant embryo.
(1.24 MB AVI)
