# Supplementary figures and images for: Loss of Myotubularin Function Results in T-Tubule Disorganization in Zebrafish and Human Myotubular Myopathy
Source: PLoS Genet. 2009 Feb 6;5(2):e1000372. doi: 10.1371/journal.pgen.1000372 (PMC2631153; doi:10.1371/journal.pgen.1000372)

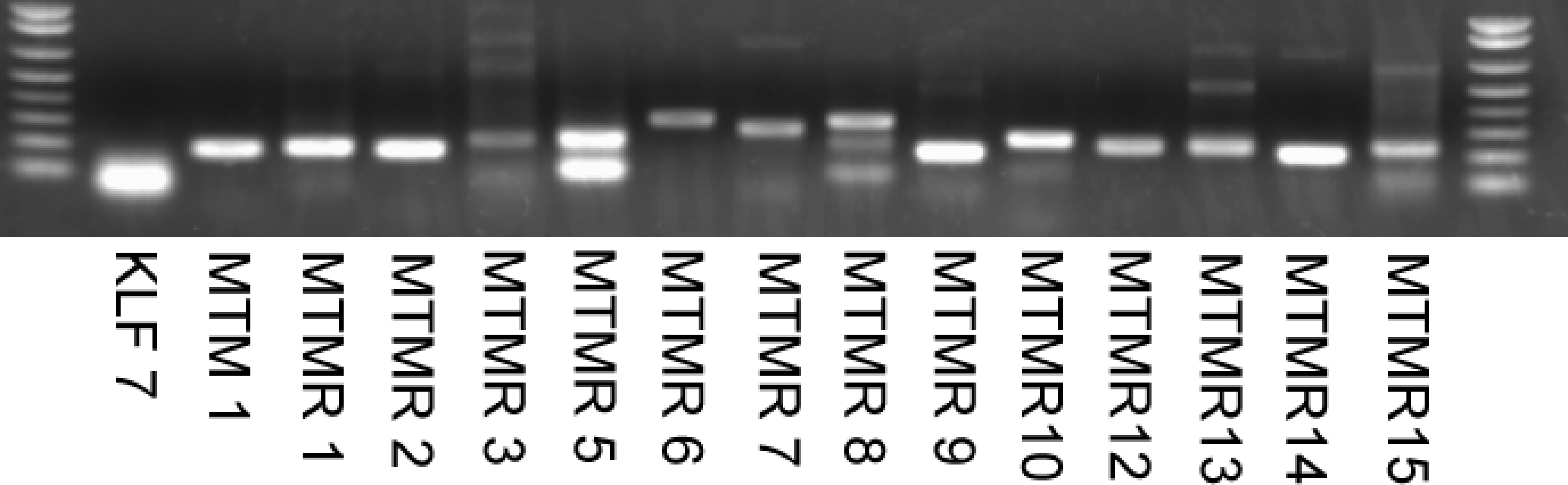

Supplement: Figure S1 — RNA expression of zebrafish MTMRs. RNA was isolated from 24 hpf zebrafish embryos and processed for RT-PCR. PCR was performed with primers specific for MTM1 and MTMRs 1–15 (excluding MTMRs 4 and 11). Bands were detected for all MTMRs tested. (0.80 MB TIF) [file pgen.1000372.s001.tif]

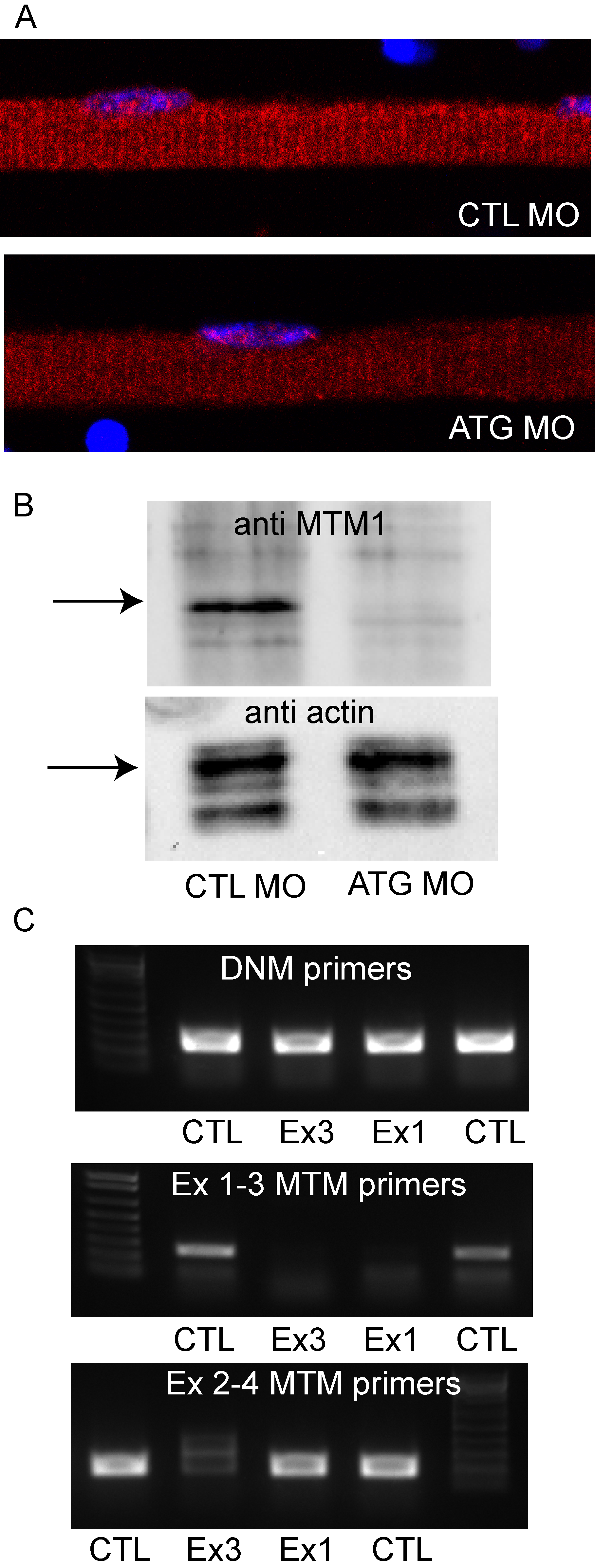

Supplement: Figure S2 — Morpholino knockdown of zebrafish myotubularin. (A) Immunohistochemistry using a myotubularin antibody on isolated myofibers from embryos injected with control (CTL MO) or myotubularin ATG (ATG MO) morpholinos. The linear staining pattern observed in control myofibers (see Figure 8) was barely detectable in MTM1 morphant fibers. (B) Western blot analysis from protein isolated from 72 hpf embryos injected with control (CTL MO) or myotubularin ATG (ATG MO) morpholinos. A band corresponding to myotubularin was detected in CTLs but not in MTM morphants. Blots were re-probed with actin to assure equal loading. (C) RT-PCR analysis from RNA extracted from 48 hpf control (CTL), exon 3 specific (Ex3), and exon 1 specific (Ex1) myotubularin morphants. Top panel: dynamin-2 (DNM) specific primers reveal intact RNA and equal starting cDNA from all samples. Middle panel: primers spanning exons 1–3 reveal reduced products for both the exon 1 and exon 3 splice blocking morphants. Bottom panel: primers spanning exons 2–4 reveal substantially reduced product in the exon 3 splice blocking morphants but a normal product in the exon 1 morphants. Taken together, the RT-PCR data reveals that the Ex1 morpholino successfully results in exclusion of exon 1 from the final RNA. The Ex3 morpholino causes exclusion of exon 3 as well as overall reduction in MTM1 RNA, likely due to nonsense mediated decay mechanisms. (5.31 MB TIF) [file pgen.1000372.s002.tif]

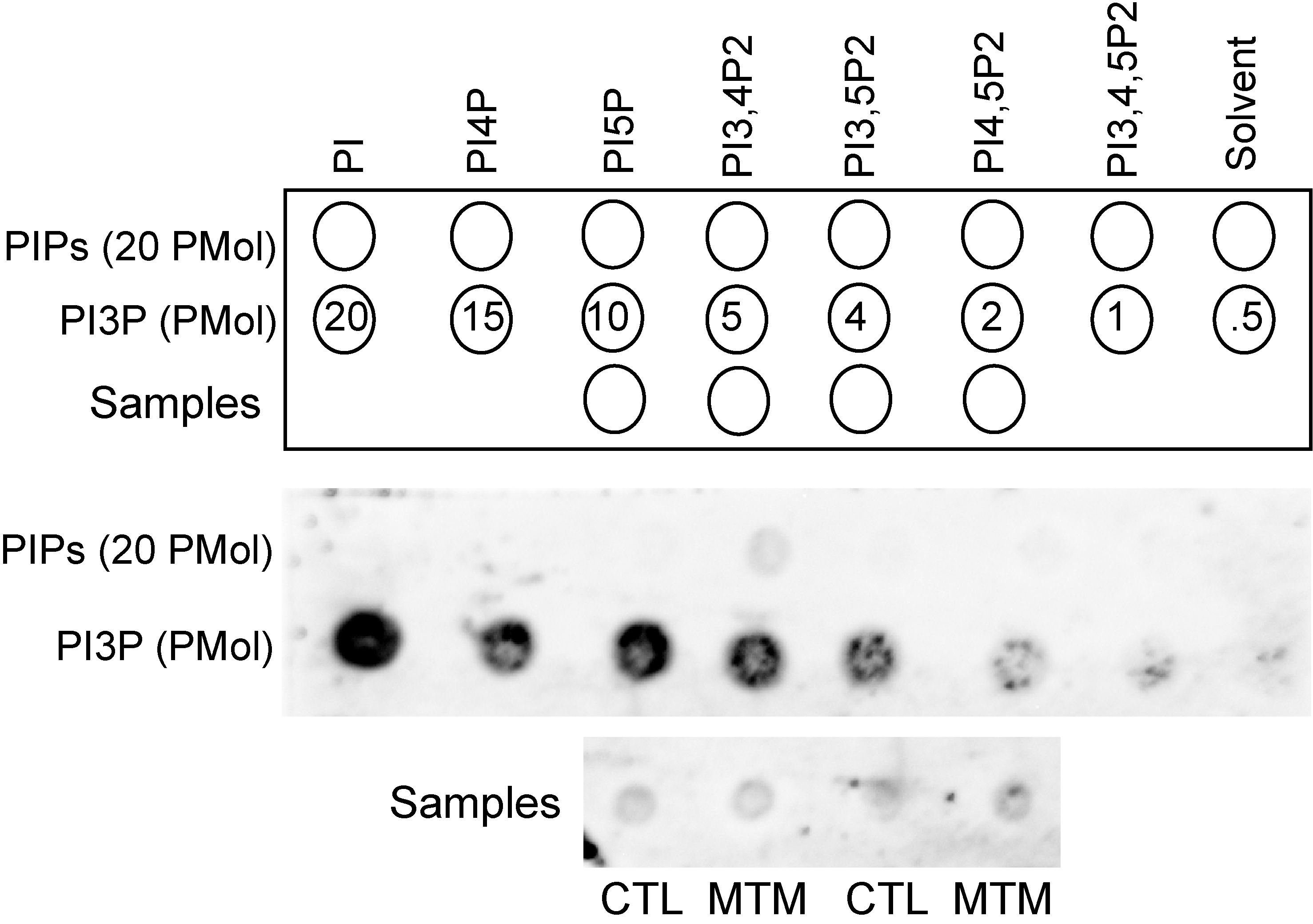

Supplement: Figure S3 — Total PI3P levels are unaffected in myotubularin morphants. PI3P mass strip assay performed on lipids isolated from control (CTL MO) or myotubularin (MTM MO) 72 hpf morphant embryos. No difference in PI3P levels was observed in MTM1 morphants (CTL 0.87 Pmol+/−0.10; MTM 0.83 Pmol+/−0.07; n = 3). Equal spotting of lipids was determined by measuring PI4P levels on duplicate samples (data not shown). (1.22 MB TIF) [file pgen.1000372.s003.tif]

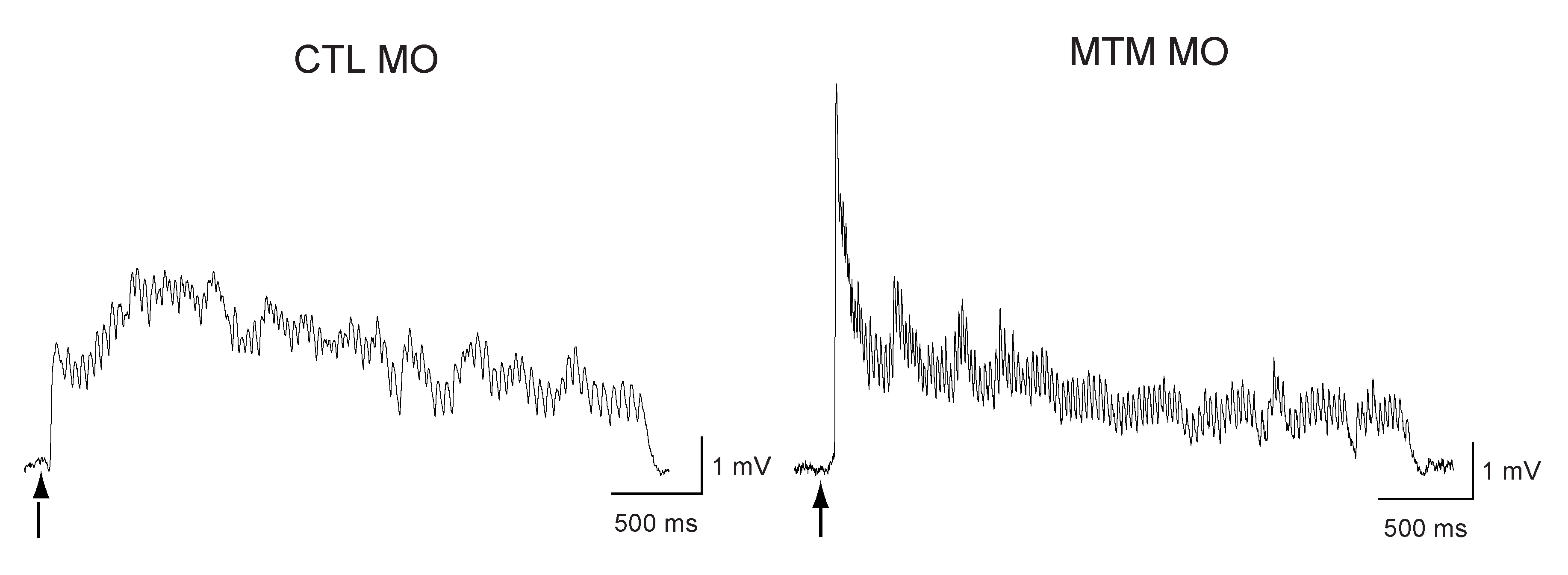

Supplement: Figure S4 — Touch-evoked fictive swimming. Touch-evoked fictive swimming in control morphants (left) and myotubularin morphants (right) in response to a 50 ms tactile stimulus. Normal responses were detected in the myotubularin morphants, indicating that neuronal input to skeletal myofibers is intact. (0.15 MB TIF) [file pgen.1000372.s004.tif]

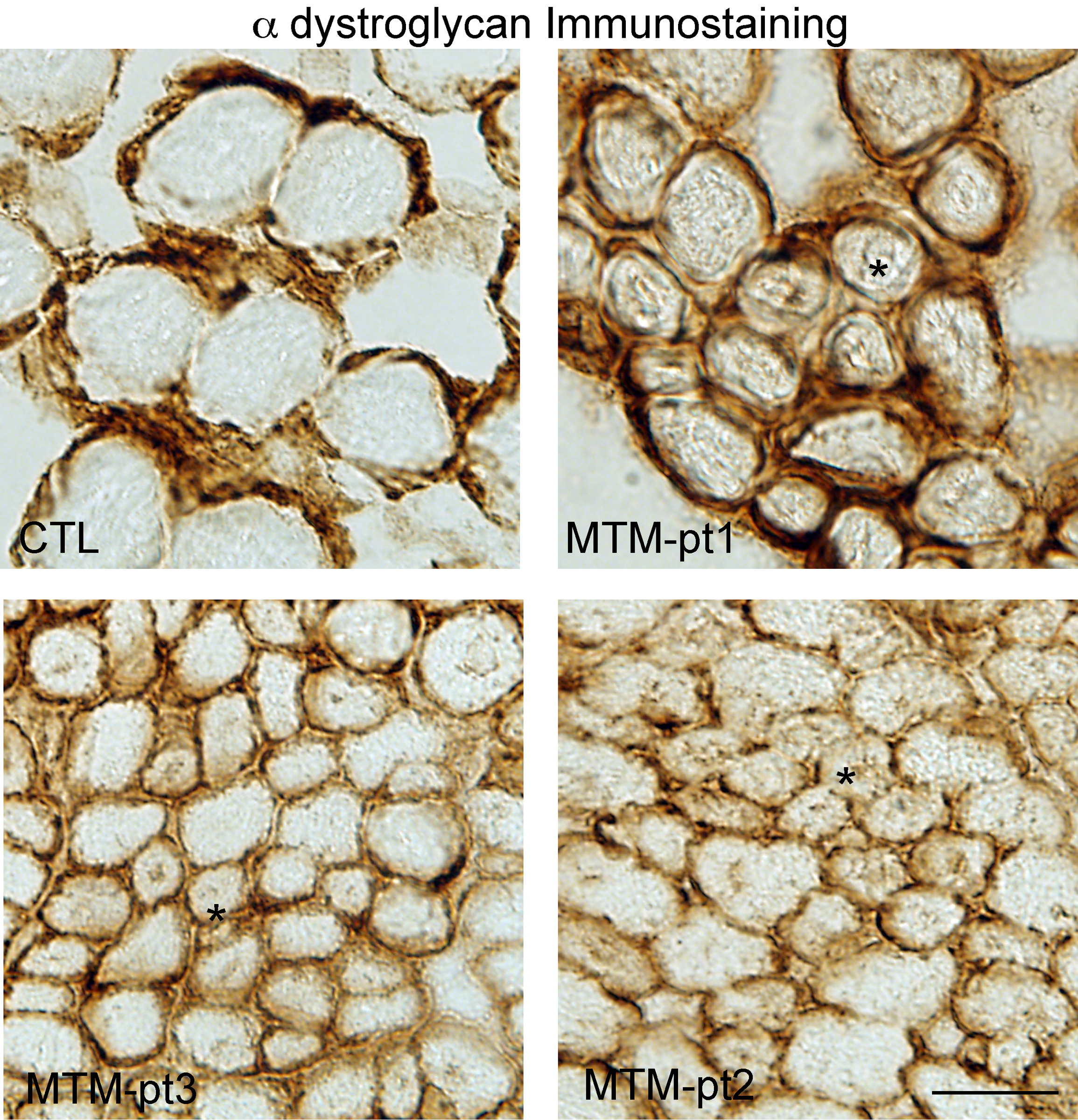

Supplement: Figure S5 — α dystroglycan staining on human biopsy samples. Immunohistochemistry with an α dystroglycan antibody. Note the normal staining around the plasma membrane and the lack of internal staining. * indicate examples of fibers with central nuclei. Scale bar = 20 mm. (9.71 MB TIF) [file pgen.1000372.s005.tif]
